# Comparative Analyses of the ****β****-Tubulin Gene and Molecular Modeling Reveal Molecular Insight into the Colchicine Resistance in Kinetoplastids Organisms

**DOI:** 10.1155/2013/843748

**Published:** 2013-09-08

**Authors:** Luis Luis, María Luisa Serrano, Mariana Hidalgo, Alexis Mendoza-León

**Affiliations:** ^1^Laboratorio de Bioquímica y Biología Molecular de Parásitos, Instituto de Biología Experimental (IBE), Facultad de Ciencias, Universidad Central de Venezuela (UCV), Caracas 1041A, Venezuela; ^2^Unidad de Química Medicinal, Facultad de Farmacia, Universidad Central de Venezuela, Caracas 1041A, Venezuela; ^3^Laboratorio de Inmunoparasitología, Centro de Microbiología, Instituto Venezolano de Investigaciones Científicas (IVIC), Caracas 1020A, Venezuela

## Abstract

Differential susceptibility to microtubule agents has been demonstrated between mammalian cells and kinetoplastid organisms such as *Leishmania spp*. and *Trypanosoma spp*. The aims of this study were to identify and characterize the architecture of the putative colchicine binding site of *Leishmania spp*. and investigate the molecular basis of colchicine resistance. We cloned and sequenced the **β**-tubulin gene of *Leishmania (Viannia) guyanensis* and established the theoretical 3D model of the protein, using the crystallographic structure of the bovine protein as template. We identified mutations on the *Leishmania* **β**-tubulin gene sequences on regions related to the putative colchicine-binding pocket, which generate amino acid substitutions and changes in the topology of this region, blocking the access of colchicine. The same mutations were found in the **β**-tubulin sequence of kinetoplastid organisms such as *Trypanosoma cruzi*, *T. brucei*, and *T. evansi*. Using molecular modelling approaches, we demonstrated that conformational changes include an elongation and torsion of an **α**-helix structure and displacement to the inside of the pocket of one **β**-sheet that hinders access of colchicine. We propose that kinetoplastid organisms show resistance to colchicine due to amino acids substitutions that generate structural changes in the putative colchicine-binding domain, which prevent colchicine access.

## 1. Introduction

*Leishmania* is the etiological agent of the human complex disease called leishmaniosis, registered as a neglected disease, which represents a public health problem with an annual incident of around 2 million people and a prevalence estimated at 12 million people worldwide [1, 2]. Until now there is no vaccine, and chemotherapy is the common treatment of the disease. Nevertheless, variation in the efficacy of treatments occurs due to, among other factors, their toxicity, differences in drug sensitivity of the parasite, acquired drug resistance, and immune compromise of the patients. The most significant advance has been the oral treatment for visceral cutaneous leishmaniosis with Miltefosine [3, 4]; evaluation of new drugs and drug combinations is in progress [3, 5]. 

Given its role in key cell processes, microtubules from different organisms, including kinetoplastids such as *Leishmania spp*. and *Trypanosoma spp.*, have been suggested as a drug target [6, 7]; the *α*- and *β*-tubulin proteins have been described as the basic components of microtubules, which are crucial for the mitotic spindle apparatus, transport, and motility. In kinetoplastids the *α*- and *β*-tubulin genes have been described as multigene families, the organization of which varies widely among species. In *Leishmania spp*. both genes have been characterized as unlinked repeats. Three *β*-tubulin loci have been identified, which are localized on chromosomes 33, 21, and 8; there is a high similarity in the coding region of the gene present in these chromosomes and the main variation that occurred upstream and downstream of the gene [8]. Polymorphism of the *β*-tubulin gene family has also been demonstrated by RFLP analysis, with sufficient variability to establish differences between both subgenera, *Leishmania* and *Viannia* [9–11].

Despite the high level of conservation and sequence identity of tubulin in eukaryotic cells, differences have been demonstrated in the susceptibility to microtubule inhibitor agents such as herbicides and vinca alkaloids. For instance, in some plants herbicide resistance has been associated to particular mutations on *α*- or *β*-tubulin [12]. Previous reports indicated differential susceptibility between mammalian cells and kinetoplastid to antimicrotubule drugs, particularly agents that bind to leishmanial tubulin [13, 14]. Of particular interest have been the different studies concerning tubulin in kinetoplastids, using cultures or purified tubulin, which revealed the ineffectiveness of colchicine or analogs and benzimidazoles on *Leishmania spp*., all potent inhibitors of mammalian tubulin polymerization [7, 14, 15]. The vinca alkaloid agents, such as vinblastine, exhibited a high effect on *Leishmania* and other kinetoplastids [15]. The isolation and purification of tubulin from kinetoplastids such as *T. brucei* [16] and *Leishmania spp*. [14, 17] have been carried out successfully, and evaluation of such preparations demonstrated their suitability for use in screening of drugs with selective antimitotic activity, such as dinitroaniline herbicides, oryzalin, and derivatives compounds [14, 18]. 

Evidence in bovids showed that the binding site for colchicine occurs in the *β*-tubulin protein [13, 19, 20]; purification and subsequent crystallography of the protein of bovine and porcine [21, 22] generated an experimental model where the identification, location, and definition of features of the colchicine binding site were possible [19, 23]. The molecular basis for the resistance to colchicine in kinetoplastids such as *Leishmania spp*. and *Trypanosoma spp*. remains incompletely understood, probably because a relationship exists between resistance and the polymorphism of the *β*-tubulin region to harbor the putative colchicine-binding site. 

This study addresses the interaction of the *β*-tubulin of *Leishmania spp.* with colchicine by comparative genomics, bioinformatics, and molecular modeling. Comparison of the experimental 3D model of bovine *β*-tubulin with the theoretical one of the *β*-tubulin protein of *Leishmania spp.* showed that resistance to colchicine is related to amino acids substitutions (AAS) in the putative binding pocket of the drug, which generated structural modifications that hinder drug access.

## 2. Materials and Methods

### 2.1. The *β*-Tubulin Gene of *L. (V.) guyanensis*: Cloning, Sequencing, and Analysis

The putative *β*-tubulin gene of *L. (V.) guyanensis*, a WHO reference* Leishmania* strain [2], was cloned as a genomic Hind III-DNA fragment into the unique Hind III site of pUC18 vector, as previously described [9, 24]. Sequencing of the *β*-tubulin DNA fragment (pLg*β*4) was conducted, using the forward and reverse universal primers of the pUC18 vector, according to standard procedures with the Sequenase Version 2.0 DNA sequencing kit (USB). Sequence analysis was performed using Basic Local Alignment Search Tool (BLAST), and multiple alignments were performed using CLUSTAL (http://www.ebi.ac.uk/tools/clustalw2) software. Protein sequences were retrieved from the Swiss-Prot database. The sequences encoding the *β*-tubulin gene used for the analysis were as follows: *L. (V.) guyanensis *(accession number ABG91756; gi: 110816092), *L. (L.) major* (accession number XP_001681156; gi: 157864895),* L. tarentolae* (accession number ABC40567; gi: 83658834), *Trypanosoma cruzi* (accession number AAL75956; gi: 18568139), *T. brucei* (accession number XP_001218933; g: 115504281), *Homo sapiens* (accession number AAB59507; gi: 338695), *Bos taurus* (accession number NP_001003900; gi: 51491829), and *Acanthamoeba polyphaga* (accession number AAZ80769; gi: 73695915). 

### 2.2. Tolerance Prediction Analysis of the Amino Acid Residues Substitution

The algorithm Sorting Intolerant From Tolerant (SIFT:http://blocks.fhcrc.org/sift/SIFT.html) was used to predict the functional effect of an amino acid substitution (AAS) according to sequence homology and the physical properties of amino acids. SIFT is based on reference sequence alignments and produces scores which can be classified as intolerant (0.00–0.05), putatively intolerant (0.051–0.10), borderline (0.101–0.20), or tolerant (0.201–1.00), according to the classification proposed previously [25–27].

### 2.3. Molecular Modeling and Structural Analysis

The homology model of the *β*-tubulin protein of *L. (V.) guyanensis* was generated using the crystallographic structure of the bovine protein (PDB 1z2B) as template [28]. An initial model was obtained with the SWISS-MODEL modeling server (http://swissmodel.expasy.org/SWISS-MODEL.html) [29] and the tools of the DeepView/Swiss-PdbViewer 4.01 software [30]. For energy refinement with NAMD, hydrogen atoms were added to the model and partial charges were assigned to all atoms. The calculations were performed with NAMD [31] using the CHARMM22 force field, with a 12 Å cut-off distance for nonbonded interactions and a dielectric constant of 1. The model was surrounded by a 12 Å water shell and energy minimized for three consecutive 10,000 conjugate-gradient steps: first, with all protein atoms fixed, second, with only the backbone atoms fixed, and third, with all atoms free. VEGA ZZ was employed to analyze the final model [32]. Molecular figures were prepared using ICM-Browser 3.6 (Molsoft L.L.C). The validation of the final model was carried out with ProSA [33] and PROCHECK programs (https://prosa.services.came.sbg.ac.at/prosa.php & http://swift.cmbi.ru.nl/servers/html/index.html) [34]. The energy of the folding, geometry, and stoichiometry of the models were evaluated by the ProSA-web and PROCHECK services (https://prosa.services.came.sbg.ac.at/prosa.php and http://www.ebi.ac.uk/thornton-srv/software/PROCHECK/). Pocket Finder was used for identification of the binding pocket (http://www.modelling.leeds.ac.uk/pocketfinder/). 

## 3. Results

### 3.1. The *β*–Tubulin Gene of *L. (V.) guyanensis*

**Figure 1:**
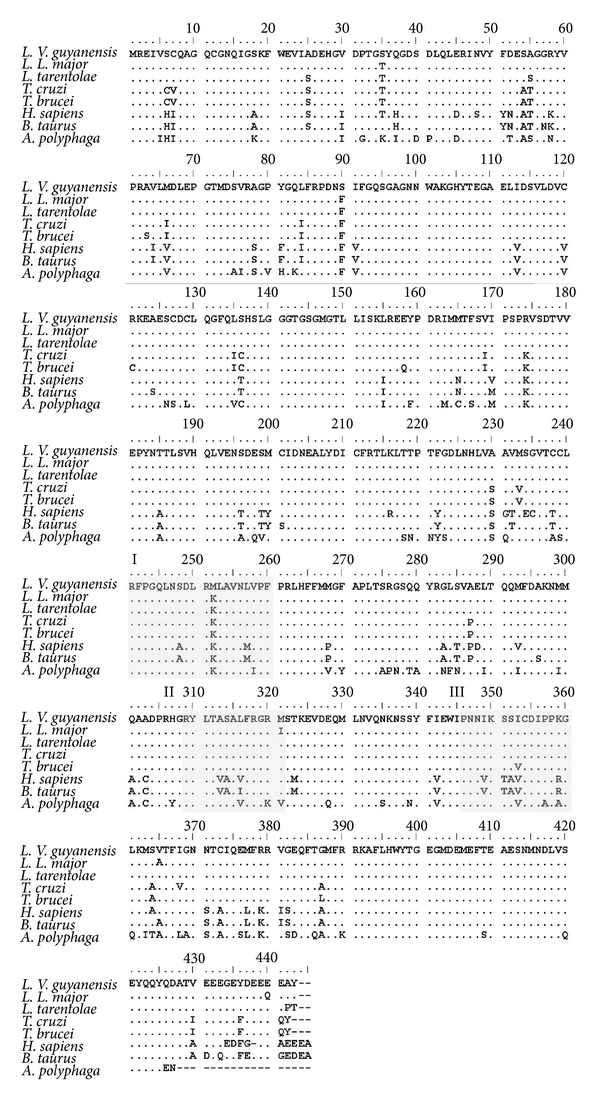
Multiple alignments of the *β*-tubulin protein of different organisms. Predicted amino acid sequences of *Leishmania (Viannia) guyanensis* M4147 (accession number ABG91756), *L. (Leishmania) major* (accession number. XP_001681156), *L. tarentolae* (accession number ABC40567), *Trypanosoma cruzi* (accession number AAL75956), *T. brucei* (accession number XP_001218933), human (accession number AAB59507), *B. taurus*. (accession number. NP_001003900), and *A. polyphaga* (accession number AAZ80769) were aligned by the CLUSTAL software. Amino acid residues involved in structural domain previously assigned to the ligands colchicine are indicated as regions I (position 240–260), II (position 309–321) and III (position 347–360).

A genomic clone, pLg*β*4, which contains the complete coding region of the *β*-tubulin gene of* L. (V.) guyanensis*, was used to obtain the nucleotide sequence [9, 24]. The gene consisted of an open reading frame of 1332 bp, which encodes a polypeptide of 444 amino acids (accession number ABG91756). Multialignment of the amino acids sequence of *L. (V.) guyanensis* with the homologous ones of other organisms available in the gene bank showed a high percent of identity (>90%) among the *β*-tubulin sequence of kinetoplastids such as *T. cruzi *(94%), *T. brucei* (93%), and other pathogenic and nonpathogenic *Leishmania*, such as *L. major* and* L. tarentolae* (98%). The analysis revealed a high conservation of the amino acid residues (Figure 1). Comparison of the *Leishmania *spp. *β*-tubulin sequence with that of bovine, human, and *Acanthamoeba* spp., a free-living amoeba which causes human keratitis, showed 82, 83, and 76% similarity, respectively. There are no introns in the total sequence of the *Leishmania spp*. gene.

### 3.2. Identification of the Putative Colchicine-Binding Region in *Leishmania spp.*

**Figure 2:**
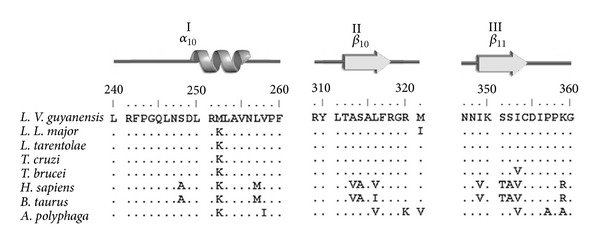
Amino acid residues sequence and secondary structure of the *β*-tubulin colchicine-binding domains. The amino acid residues sequence of the *β*-tubulin colchicine-binding domains I, II, and III of different organisms were aligned and compared to visualize amino acid substitutions (AAS) generated by single nucleotide mutations; eleven AAS were labeled and cited in the text and Table 1. Identical amino acid residues are indicated by points.

In order to identify the putative colchicine binding domains, we analyzed the primary structure of the *β*-tubulin protein of *Leishmania spp*. Similar to bovine (*B. taurus*), used as reference organism, *Leishmania spp*. showed three regions which comprise several amino acid residues: region I from 240 to 260, region II from 309 to 321, and region III from 347 to 360 (Figure 1). Comparison of the primary structure of these regions with *B. taurus* showed a total of eleven amino acid substitutions (AAS) in *Leishmania*, including the *Sauroleishmania L. tarentolae* (Figure 2; Table 1). Probably, the more important ones were four of these changes due to nonsynonymous mutations (nsSNP), which generated AAS; three of them involve the nonpolar amino acid Ala (A) present in bovine at positions 248, 314, and 352, respectively, which was changed in *Leishmania* for the polar amino acid Ser (S); the fourth mutation generated a change of the basic amino acid Lys (K) to the non-polar one Met (M) at position 257. Seven of the eleven mutations in* Leishmania,* including those at positions 248, 314 and 352, were also found in the *β*-tubulin sequence of *A. polyphaga*, an organism which shows colchicine resistance (Figure 2). 

Once the above amino acid changes in *Leishmania spp*. were determined, we carried out an analysis of tolerance prediction of the AAS on the colchicine-binding region of bovine, which was used as reference organism, using the SIFT program (see Section 2). The analysis showed that only four synonymous mutations, of the eleven present on the *β*-tubulin of *Leishmania*, could be tolerated for bovine protein (PDB 1z2B); these included those at positions ^257^Met-^257^Leu (^257 ^M→^257^L), ^313^Val-^313^Ala (^313^V→^313^A), ^316^Val-^316^Leu (^316^V→^316^L), and ^353^Val-^353^Ile (^353^V→^353^I), whereas the other seven mutations were not tolerated for bovine protein (Table 1). The nonsynonymous mutations in positions ^248^Ala-^248^Ser (^248^A→^248^S), ^252^Lys-^252^Met (^252 ^K→^252 ^M), ^314^Ala-^314^Ser (^314^A→^314^S), and ^352^Ala-^352^Ser (^352^A→^352^S) apparently generate structural modification in the properties of *Leishmania* putative colchicine binding pocket.

### 3.3. Modeling of the *β*-Tubulin Protein and Structural Analysis of the Putative Colchicine-Binding Region

**Figure 3:**
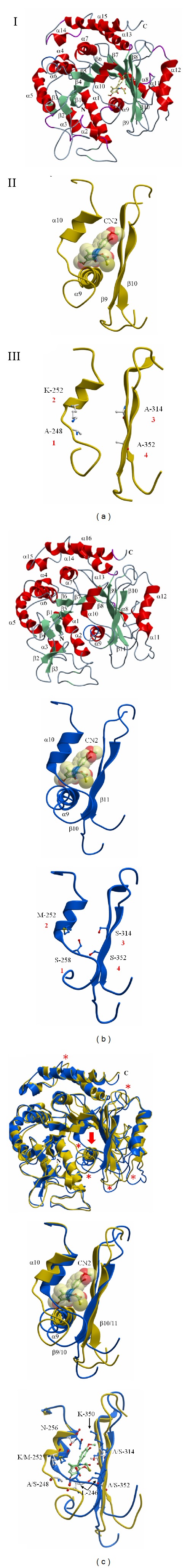
The conformation of the colchicine-binding domain on the *β*-tubulin protein. (I) Comparison of the ribbon diagram of the crystallography model of the *β*-tubulin protein of bovine (a) and the homology model of *L. (V.) guyanensis* (b). Secondary structure elements (*α* helix, *β*-sheet, and coil) and the N and C terminal are marked. Overlay of both structures (yellow: bovine; blue: *Leishmania*) is presented (c). Arrow indicates the colchicine-binding domain; regions showing significant differences are marked (*). (II) Overview of the conformation of the colchicine-binding domain of the *β*-tubulin protein of bovine (yellow) and *Leishmania *(blue). The ligands colchicine (CN2) bound to the domain and secondary structure elements associated with this region are presented and defined as follows: helix *α*_9-10_ and *β*_9-10_ sheet. The CN2 bound to the putative domain of *Leishmania* corresponds to a hypothetical representation to visualize the conformational changes in comparison with the bovine region. Superimposition of both structures is presented. (III) Amino acids residues substitutions and structural changes. The colchicine domains without ligands are the same as (II). The nonsynonymous mutation at positions 248 (1), 252 (2), 314 (3), and 352 (4), which generated change of nonpolar amino acid residues Ala (A), and the basic Lys (K) present in bovine to the polar one Ser (S) and nonpolar Met (M) in *Leishmania* are indicated. The side chain of particular amino acid residues, such as S and common amino acid residues Asn (^256^N) and Lys (^350 ^K), embedded inside the *Leishmania *colchicine domain is presented on the superimposed of these structures, including CN2.

In order to evaluate the structural features of the putative binding pocket of colchicine of the *β*-tubulin of *L. (V.) guyanensis*, we generated a theoretical 3D model by the SWISS-MODEL package, based in sequence identity (83%) and using the experimental crystallography model of the bovine protein (PDB 1z2B) as template. Comparison of bovine (Figure 3I(a)) with the homology model of *Leishmania spp*. (Figure 3I(b)) suggested a similar structure for the protein in these organisms. The quality of the model was established by superimposition on the crystal structure of the template, both structures showing high similarities; however, differences were evident between them (Figure 3I(c), asterisks). The estimated value of root mean-square deviation (rmsd) was 2.01 Å for the C*α* atoms (Table 2), suggesting deviation of our model from the template.

### 3.4. Conformation of the Putative Colchicine-Binding Pocket

In order to explain the occurrence of potential conformational changes, the theoretical model being established, we evaluated the secondary structure and orientation of amino acid side chains involved in the binding of colchicine. A comparison of the colchicine binding pocket of bovine and the putative one of *Leishmania*, showing the peptide backbone as a ribbon diagram where colchicine (CN2) is visible, revealed structural differences related to secondary structure elements of the *β*-tubulin protein. The main feature was the presence in *Leishmania* of an elongation of an *α*-helix (*α*_9-10_) structure oriented toward the inside of the pocket and a torsion thereof, which is not present in bovine (Figures 3II(a) and 3II(b)); also, one of the two *β*-sheet (*β*_9_) secondary structures of the *Leishmania* pocket has a displacement towards the inside of the pocket. The superimposition of both structures (Figure 3II(c)) showed that these new features probably cause topological alterations that prevent colchicine access. 

 As mentioned, comparison of the amino acid residues in the colchicine pocket of bovine (Figure 3III(a)) with those identified in the putative pocket in *Leishmania* (Figure 3III(b)) showed several single nucleotide mutations, some of them generating AAS. The overlay of both structures showed that these AAS directly produce topological modifications. For example, the substitutions ^248^A→^248^S (nonpolar to polar amino acid residues) and ^349^V→^349^I (nonpolar to nonpolar amino acid residues) result in side chains, of some amino acid residues, becoming embedded inside the putative colchicine pocket with local topology changes, which produces a reduction of the space available for the ligand (Figure 3III(c)). 

## 4. Discussion

We have used a modeling approach to address the conformational changes in the putative colchicine-binding site of the *β*-tubulin of *Leishmania spp.* to explain the lack of susceptibility of *Leishmania* and other kinetoplastid to colchicine. First of all, we established the *β*-tubulin gene sequence of *L. (V.) guyanensis* and, by comparative analysis with homologous sequences from different organisms, we established the high conservation of the protein among both pathogenic and nonpathogenic *Leishmania*, such as *L. tarentolae*. Similar results were found when comparison was carried out with other kinetoplastids, such as *T. cruzi*, *T. brucei,* and *T. evansi* [35]. There is sufficient polymorphism at the nucleotide level to explain the differences previously demonstrated among *Leishmania* subgenera; the region harboring the *β*-tubulin genes showed sufficient variability to be used as a molecular marker [10, 24].

An analysis of the primary structure of the *β*-tubulin protein of *Leishmania spp*. using as reference the colchicine binding domains of bovine and porcine [19, 20, 22, 36], which are similar to human protein and also sensitive to colchicine, identified synonymous (sSNP) and nonsynonymous (nsSNP) single nucleotide substitutions on the putative colchicine binding region of* Leishmania spp.*, including *L. tarentolae*. Such mutations generated a total of eleven AAS related to drug resistant. Noteworthy, the same mutations were present in the homologous gene of other kinetoplastids, such as *T. cruzi*, *T. brucei,* and* T. evansi* [35]. Since some of these mutations are tolerated for the tubulin region of bovine according to the tolerance test, we concluded that not all mutations identified on the *β*-tubulin protein of* Leishmania* ssp. and other kinetoplastids have an identical contribution to colchicine resistance. Interestingly, seven of these mutations were present in different species of *Acanthamoeba*, also resistant to colchicine [37], supporting the important role of these changes in the resistance phenotype of *Leishmania spp*. and other kinetoplastids. 

 Different isotypes for *β*-tubulin are present in human cells and overexpression of some of these isotypes, such as *β*I and *β*III-tubulin, are associated with resistance to tubulin-binding agents in some cancer cells [6, 36, 38, 39]. Based on the primary amino acids sequences, available in the gene bank, from the *β*-tubulin of *Leishmania spp*. and other kinetoplastid organisms mentioned in this work for comparison, isotypes variability or differential isotype expression does not seem to explain the colchicine resistance, since there is no a high variability in the domains identified as the putative colchicine-binding pocket in these organisms [8, 35]. A recent study in cancer cells, using computational modeling, showed the relationship between mutations in the tubulin isotypes *β*I and *β*III and the resistance to microtubules disruptor compounds [40, 41]. 

 No structural *β*-tubulin model for *Leishmania spp*. has been published so far; we have successfully established the structural homology model of the *β*-tubulin protein of *L. (V.) guyanensis*, which gave us the opportunity to identify and establish the features of the putative-binding site for the antimitotic colchicine drug. The *Leishmania* model showed folding pattern similarities, compatible with the crystallographic structure of the bovine *β*-tubulin protein (83% sequence identity) that was used as template. A *z*-score value of −8.87 for the structural model of *L. (V.) guyanensis* suggested typical features of a native structure; however, the estimated value of rmsd for the C*α* atoms suggested deviation of our model from the template, probably due to important conformational changes. In fact, we found that such conformational changes were associated to the domains identified as the putative colchicine-binding pocket; the elongation and torsion of an *α*-helix (*α*_9_) structure and the displacement of the *β*-sheet (*β*_10_) prevent this region from adopting a proper molecular architecture conformation to harbor colchicine. These features were absent in bovine protein. The above interpretation could help explain the ineffectiveness of colchicine demonstrated through assays, where drug effects were determined through quenching of fluorescence emission or reduction in sulfhydryl reactivity [14].

 Some non-tolerate mutations generate particular AAS on the putative colchicine pocket of *Leishmania ssp*., embedding side chains of other amino acid residues inside the cavity, which probably generate local topological changes, such as a volume reduction of the space available to the ligand. In fact, the volume estimation of the colchicine-binding cavity in bovine and further comparison with that of *Leishmania ssp*. support this hypothesis. A volume of 518 Å^3^ for the bovine colchicine pocket was found, whereas, the colchicine cavity in *Leishmania spp.* seems to be absent. Alternatively, the structural architecture of the cavity in the putative binding pocket may not be display. Apparently, the polarity features of the *Leishmania spp*. region produce important physicochemical changes. 

 In conclusion, this is the first report on the features of the colchicine-binding domain in the *β*-tubulin protein of *Leishmania spp.* or other kinetoplastids. Analysis of the primary structure of the protein showed eleven AAS, which are related with colchicine resistance. In addition, we have established the first theoretical 3D model of the *β*-tubulin protein of *Leishmania spp.,* and comparison of the experimental bovine and porcine models suggests that colchicine resistance is due to structural changes generated by the AAS in the putative binding domain of the drug, which prevent colchicine access. The AAS resulted in side chains of the neighboring amino acids going inside the pocket contributing with additional topological changes also, which prevent this region from adopting an appropriate molecular architecture conformation of the cavity. We generalize that the molecular basis for colchicine resistance in kinetoplastids is due to AAS in the putative binding domain of the drug, which generates structural modification that hinder drug access. Further work using molecular dynamics and docking approaches is in progress, in order to define whether this region could be exploited as a potential specific target and, also, to identify compounds that selectively interact with this colchicine binding like *β*-tubulin region of *Leishmania spp*.

## Figures and Tables

**Table 1:** Tolerance prediction analysis of the amino acid residues substitution on the colchicine-binding domain.

ns/s SNP* Position	AAS^§^			Scores^#^	Impact^¶^
	Bovine	*A. polyphaga *	*Leishmania *		
248	A (np)	S	S (p)	0.01	NT
252	K (b)	K	M (np)^+^	0.00	NT
257	M (np)	L	L (np)	0.06	T
313	V (np)	A	A (np)	1.00	T
314	A (np)	S	S (p)	0.04	NT
316	V (np)	V	L (np)	0.10	T
349	V (np)	I	I (np)	0.02	NT
351	T (p)	S	S (p)	0.00	NT
352	A (np)	S	S (p)	0.00	NT
353	V (np)	V	I (np)^#^	0.05	T
359	R (b)	A	K (b)	0.01	NT

*ns SNP: non-synonymous single nucleotide polymorphisms; sSNP: synonymous SNP. ^§^AAS: amino acids substitution; p, np and b: polar, non-polar and basic amino acid. ^#^Score range was established among bovine and *Leishmania* sequences. ^¶^T: tolerant; NT: non-tolerant. ^+^This amino acid residue is only present in *L. (V.) guyanensis*. ^#^This amino acid residue is not present on both, *T. brucei* or *T. evansi.* The algorithm Sorting Intolerant From Tolerant (SIFT) was used as described in Material ad Methods. The indicate value are related with the tolerance (T) and non-tolerance (NT) of the amino acids substitutions, according to SIFT scores: non-tolerant (0.00–0.05), putatively intolerant (0.051–0.10), borderline (0.101–0.20), or tolerant (0.201–1.00) (25, 26, 39). Amino acid categories: nonpolar (np), polar (p) and basic (b).

**Table 2:** Structure comparison of the *β*-tubulin model of *L. (V.) guyanensis* and *Bos taurus. *

	*L. (V.) guyanensis *	*Bos taurus *
PDB Code	—	1Z2B
Number of residues	426	418
Sequence identity with *L. (V.) guyanensis *	—	84%
msd C_*α*_ versus *L. (V.) guyanensis *	—	2.01 Å (418 C_*α*_ atoms)
Total area Å^2^	18592.2	16732.0
(Total volume Å^3^)	(36286.5)	(36262.4)
%Phi/Psi^¶^	78.6	73.3

PDB: Protein Data Bank code; rmsd: root-mean-square deviation; C_*α*_: *α* carbon. ^¶^Phi and Psi are torsion angle of the all residues of the *L. (V.) guyanensis* chain, %Phi/Psi: Phi-Psi combination in favourable regions of the Ramachandran plot, *Z*-score: −8.87.
